# RNA Polymerase II Pausing Downstream of Core Histone Genes Is Different from Genes Producing Polyadenylated Transcripts

**DOI:** 10.1371/journal.pone.0038769

**Published:** 2012-06-11

**Authors:** Krishanpal Anamika, Àkos Gyenis, Laetitia Poidevin, Olivier Poch, Làszlò Tora

**Affiliations:** 1 Department of Functional Genomics and Cancer, Institut de Génétique et de Biologie Moléculaire et Cellulaire (IGBMC), CNRS UMR 7104, INSERM U 964, Université de Strasbourg, Illkirch, France; 2 Department of Structural Biology and Genomics, Institut de Génétique et de Biologie Moléculaire et Cellulaire (IGBMC), CNRS UMR 7104, INSERM U 964, Université de Strasbourg, Illkirch, France; Karlsruhe Institute of Technology, Germany

## Abstract

Recent genome-wide chromatin immunoprecipitation coupled high throughput sequencing (ChIP-seq) analyses performed in various eukaryotic organisms, analysed RNA Polymerase II (Pol II) pausing around the transcription start sites of genes. In this study we have further investigated genome-wide binding of Pol II downstream of the 3′ end of the annotated genes (EAGs) by ChIP-seq in human cells. At almost all expressed genes we observed Pol II occupancy downstream of the EAGs suggesting that Pol II pausing 3′ from the transcription units is a rather common phenomenon. Downstream of EAGs Pol II transcripts can also be detected by global run-on and sequencing, suggesting the presence of functionally active Pol II. Based on Pol II occupancy downstream of EAGs we could distinguish distinct clusters of Pol II pause patterns. On core histone genes, coding for non-polyadenylated transcripts, Pol II occupancy is quickly dropping after the EAG. In contrast, on genes, whose transcripts undergo polyA tail addition [poly(A)^+^], Pol II occupancy downstream of the EAGs can be detected up to 4–6 kb. Inhibition of polyadenylation significantly increased Pol II occupancy downstream of EAGs at poly(A)^+^ genes, but not at the EAGs of core histone genes. The differential genome-wide Pol II occupancy profiles 3′ of the EAGs have also been confirmed in mouse embryonic stem (mES) cells, indicating that Pol II pauses genome-wide downstream of the EAGs in mammalian cells. Moreover, in mES cells the sharp drop of Pol II signal at the EAG of core histone genes seems to be independent of the phosphorylation status of the C-terminal domain of the large subunit of Pol II. Thus, our study uncovers a potential link between different mRNA 3′ end processing mechanisms and consequent Pol II transcription termination processes.

## Introduction

RNA polymerase II (Pol II) transcription is a highly regulated process that requires coordinated action of numerous transcription factors. It can be divided into initiation, promoter escape, elongation and termination phases. Transcription initiation is a complex series of ordered multistep process that involves the recruitment of Pol II to a promoter, local melting of the DNA around the transcription start site (TSS), and formation of the first few phosphodiester bonds of mRNA. Recognition of promoters begins with the assembly of a large protein complex, called the preinitiation complex (PIC), containing Pol II and multiple general transcription factors (GTFs) on the promoter [1]. Recruitment of Pol II to eukaryotic gene promoters by specific transcription factors is a key regulatory step in transcription initiation [2], [3].

Several studies analysed Pol II binding at the promoter and its entry in transcription genome-wide by using chromatin immunoprecipitation (ChIP) assay coupled either to genomic DNA microarrays (ChIP-chip) or to high throughput sequencing (ChIP-seq). These reports uncovered a significant fraction of genes that have high levels of Pol II density at or around their TSSs when compared to the transcribed region of the genes [4], [5], [6], [7], [8], [9], [10]. Many of these genome-wide experiments demonstrated that these high peaks, reflecting Pol II binding accumulation at promoters, were centered around a region 20–50 nucleotides downstream of the TSSs of the genes. Thus, it is now accepted that these high Pol II densities or occupancy signals reflect Pol II pausing at promoter-proximal sites of many transcribed, but also non-transcribed, genes in different organisms. Promoter proximal pausing (PPP) of Pol II was first described at the *Drosophila* heat shock gene (*HSP70*) promoters, and shown that Pol II stalls 20–50 nucleotides downstream from the TSS [11], [12]. PPP was once considered a rare phenomenon, but the recent genome-wide reports have demonstrated that it is a common and widespread regulatory step in eukaryotic Pol II transcription [6], [7], [8], [9], [13]. In some of these earlier analyses, Pol II pausing has been observed at the 3′ end of the genes however none of these genome-wide Pol II binding studies analysed and characterized in details Pol II binding and pausing around the 3′ of the transcription units.

It is long established that 3′-end processing is absolutely required for transcription termination [14]. Transcription termination is defined as the cessation of RNA synthesis and release of Pol II from its DNA template [15]. The analysis of transcription termination event carried out on few model genes showed that termination is dependent on polyadenylation (polyA) signals and downstream terminator sequences [16]. Pol II has been suggested to terminate transcription at sites positioned between 100 bp and several kbps downstream of the 3′ end of the annotated genes (EAGs) [17], [18], [19], [20], [21]. Downstream of the EAGs two classes of terminator sequences have been identified in human genes: G-rich transcription pause sites and co-transcriptionally cleaved (CoTC) RNA sequences [16], [21], [22], [23]. Earlier reports suggest a link between transcription termination and 3′ end processing of Pol II transcripts in which two general models were put forward: the “allosteric” or “anti-terminator” model and the “torpedo” model [24], [25]. The allosteric model proposes that the presence of a polyadenylation sequence on the RNA triggers a change in the factors associated with the polymerase [26]. In this model, binding of the cleavage and polyadenylation specificity factors (CPSFs) and the cleavage stimulation factors (CstFs) to the AAUAAA polyadenylation signal on the nascent pre-mRNA favors transcription termination by displacing elongation factors and consequently rendering Pol II less processive [24], [27], [28], [29]. In the torpedo model, the cleavage event at the polyadenylation site generates a new 5′ end [30]. Unlike the capped 5′ end of the pre-mRNA, this extremity could act as an entry point for an activity (such as Xrn2 exonuclease or a helicase) that would track along the RNA and dissociate the polymerase from the DNA template. A recent work has shown that the 5′–3′ exonuclease Xrn2 in human and Rat1 in yeast are required for efficient termination [31], [32]. The 3′-end processing coupled termination of Pol II by Xrn2-mediated torpedo effects are established for poly(A)^+^ genes, but only inferred for histone genes [14]. However, Luo et al. (2006) reported that neither the “anti-terminator” nor the “torpedo” model is sufficient to cause Pol II termination, instead a termination mechanism, which is a hybrid of the two models, may exist [33].

Besides the existence of a link between pre-mRNA 3′ end processing and termination, Pol II pausing has also been implicated in promoting transcription termination. Studies in mammalian cells indicate that termination can be separated into two steps: pausing and polymerase release [34]. Recently, a study performed on the β-globin/β-actin reporter gene suggested that R-loops formed by DNA/RNA hybrids near G-rich pause sites, downstream of polyA signals may be involved in transcription termination [35]. In contrast, other studies argue that Pol II pausing is exclusively a function of polyadenylation signals and does not require any additional elements or pausing sites in the DNA sequence [36], [37], [38], [39]. In mammalian systems, the main focus of genome-wide study with respect to pausing was PPP and pause/stalling at or 3′ of EAGs has been studied just for few individual genes [40], [41]. However, the full spectrum of genes regulated by pausing and different pausing profiles at or downstream of EAG has not yet been investigated at a genome-wide level. Thus, it is not clear whether this phenomenon is commonly occurring among mammalian genes.

To characterize Pol II pausing downstream of the EAG of the transcription units in human MCF7 cells genome-wide, we used high-resolution occupancy profiling by ChIP-seq of Pol II in human cells. Comparison of our ChIP-seq results with (i) recently published Gro-seq data [42], where the authors mapped genome-wide the position, amount, and orientation of transcriptionally engaged Pol II in MCF7 cells, and (ii) published ChIP-seq data from mouse embryonic stem cell (ESCs) with several different forms of Pol II [5], [43] indicates that active Pol II pauses genome-wide downstream of the EAGs in mammalian cells. Interestingly, Pol II occupancy downstream of the EAGs can be detected up to 6 kb from the EAGs, but core histone genes, which encode non-polyadenylated transcripts, show a very narrow Pol II pause downstream of their EAGs.

## Results

### The accumulation of both Pol II and the corresponding transcripts downstream of the EAGs is a genome-wide feature of Pol II transcription

**Figure 1:**
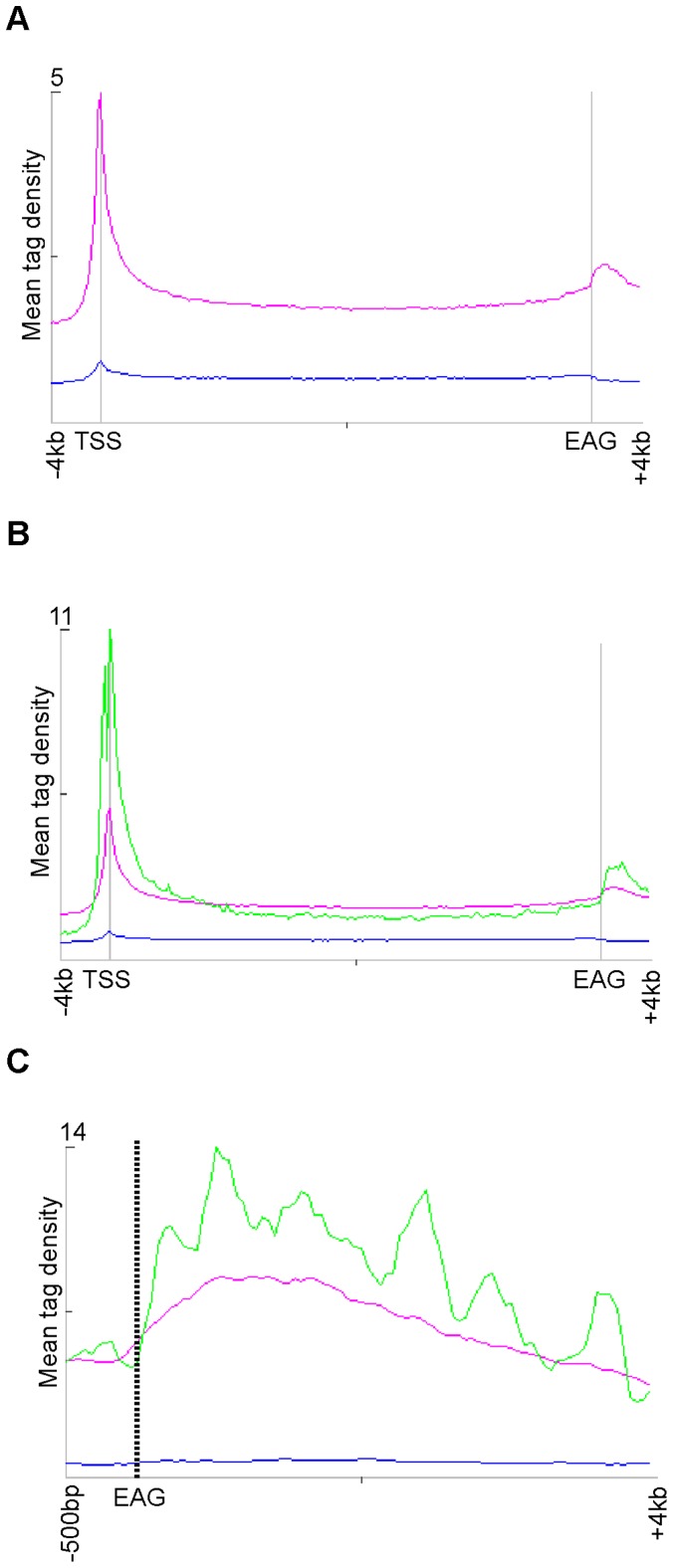
Pol II pausing and the corresponding transcripts downstream of the EAGs are genome-wide features of Pol II transcription termination. Chip-seq data using an anti-Pol II antibody (N-20, our study) and Gro-seq [42] was carried out using human MCF7 cells. **A**) Mean tag densities of ChIP-seq data of Mock (Blue) and Pol II (Pink) in average genes and −/+4 kb around them are represented. Pol II enrichment density of 13787 “non-overlapping and isolated” refseq genes were calculated. TSS: transcription start site; EAG: end of annotated gene. **B**) Mean tag densities of Mock (Blue) and Pol II ChIP-seq (Pink) and Gro-seq (Green) data on average 13787 “non-overlapping and isolated” refseq genes, and −/+4 kb upstream and downstream are represented. **C**) Mean Pol II tag density from the ChIP-seq of Mock and Pol II (Blue and Pink, respectively) and Gro-seq (Green) data in the region −500 bp to +4 kb around the EAG of 500 highly expressed genes (Table S1). Note that all the Gro-seq RNA reads map in the sense orientation when compared to the pre-mRNA.

To better understand Pol II behavior at the 3′ end of the transcription units and in transcription termination, we have analysed the patterns and profiles of Pol II binding downstream of EAGs in differentiated human cells. To this end, we have generated ChIP-seq data for Pol II from the MCF7 human breast cancer cell line (GSE34001) by using an antibody that binds to the N-terminus of the largest subunit of Pol II (N-20; Santa Cruz, H-224X). This antibody allows the detection of Pol II independently of the phosphorylation status of the C-terminal domain (CTD) of its largest subunit. To test for non-specific binding, we carried out a control ChIP-seq using an antibody raised against a yeast factor that does not recognize any human proteins (Mock; GSE34001). Sequencing reads were mapped to human genome, and uniquely mapped reads were considered for further analyses. To avoid the overlapping of Pol II occupancy signals downstream from the EAGs with signals coming from neighboring transcription units, only those human refseq genes (13787 genes) were analysed, which were at least 4 kb away from the neighboring transcription units. Pol II tag density −/+4 kb around the gene body and in the transcribed regions was then calculated. The average tag density calculated on 13787 genes, which do not have genes in −/+4 kb neighboring region, shows that Pol II binding profiles are relatively low in the transcribed regions, but higher at both, around the TSSs and 3′ from the EAGs (Figure 1A). These data suggest that genome-wide Pol II pauses not only at the TSSs, as previously reported [40], but also downstream of the EAGs (Figure 1A). Furthermore, a comparison of the Pol II ChIP-seq data with publicly available Gro-seq data demonstrates that transcriptionally engaged Pol II is present not only in the transcription units [42], but also downstream of the EAGs (Figure 1B). The genome-wide comparison of both, the Pol II occupancy and the corresponding transcript production mapped by Gro-seq around the EAGs (−500 bp to +4 kb from EAG; Figure 1C) of 500 highly expressed genes (Table S1) shows that indeed transcriptionally active Pol II is bound in the regions downstream from the EAGs. Interestingly, in these regions all the transcripts identified by Gro-seq are mapping in the sense orientation 3′ from the EAGs suggesting that they are produced by Pol II molecules that have been transcribing pre-mRNAs. As observed previously, by analyzing the transcriptionally engaged Pol II obtained by Gro-seq [44], we did not detect any significant antisense transcription 3′ from the EAGs of the genes. Taken together our analysis, in good agreement with previous studies [5], [9], [44], suggests that the accumulation of Pol II downstream of the EAGs is a genome-wide feature of Pol II transcription.

### Different patterns of genome-wide Pol II pausing downstream of the EAGs

**Figure 2:**
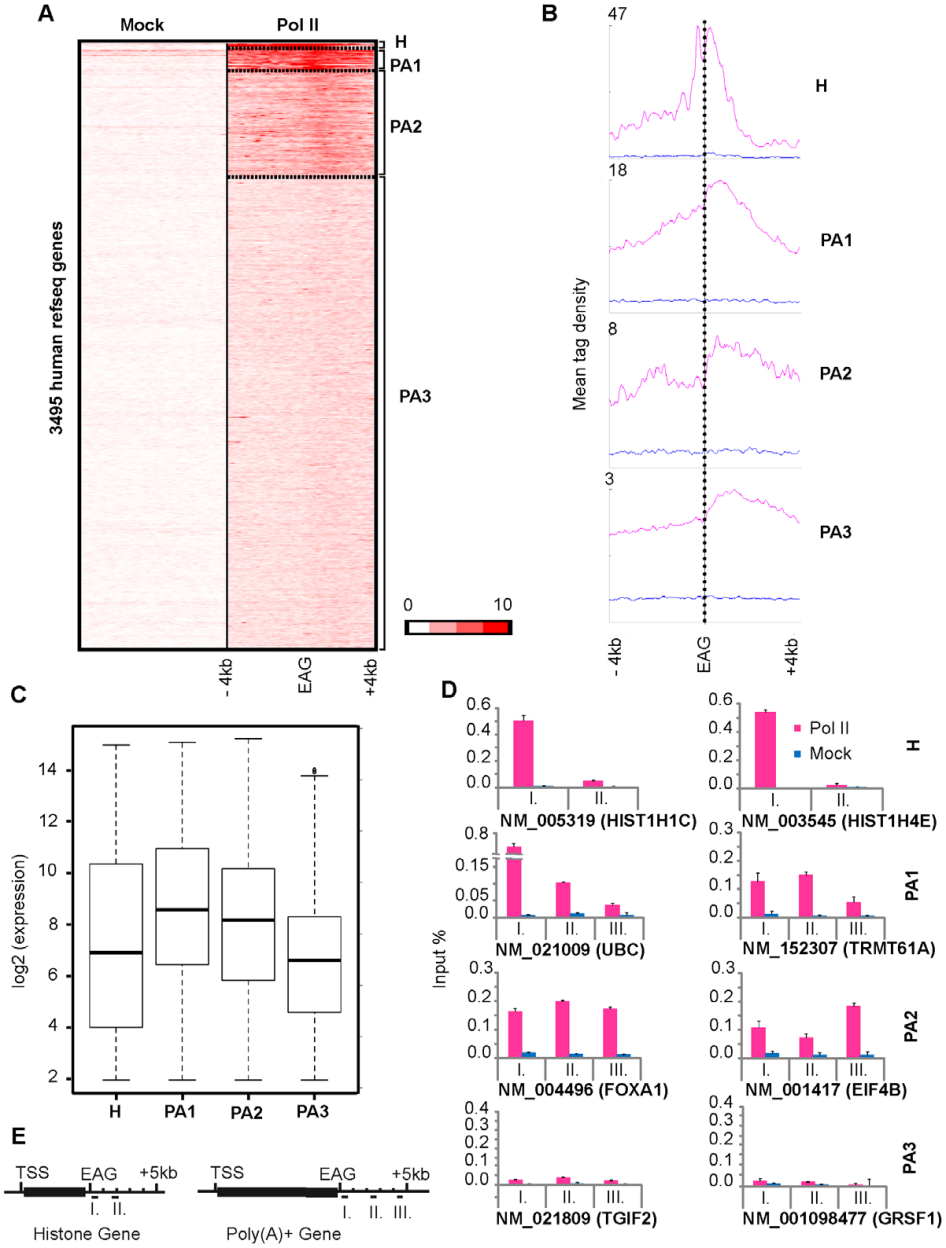
Genome-wide Pol II pauses with different patterns downstream of the EAGs. Clustering of genes, which have relatively high Pol II enrichment 3′ of their EAGs and high microarray expression value (considering genes from Cluster 1 of Figure S1) generates four clusters: H, PA1, PA2 and PA3. Total number of non-redundant refseq genes is 3495. Number of genes (or n) in each cluster is: Cluster H, n = 39; Cluster PA1, n = 74; Cluster PA2, n = 492 and Cluster PA3, n = 2890. **A**) Heatmap generated after K-means clustering of Mock and Pol II reads in the regions −/+4 kb upstream and downstream of the EAG. Color scale indicates the level of enrichment. **B**) Mean tag densities of Mock (Blue) and Pol II (Pink) on genes −/+4 kb upstream and downstream of the EAG in each cluster. **C**) The distribution of the expression levels of genes belonging to the H, PA1, PA2 and PA3 clusters (see A and B) is displayed by Whisker plot. The plots represent relative mRNA expression level of each cluster. The median is indicated with a horizontal line in each box showing that genes in Cluster PA1 have higher relative mRNA expression level than Cluster PA2 and PA3. **D**) ChIP-qPCR validation of the ChIP-seq data on two randomly selected genes (refseq ids and gene names are given) from each cluster. Pol II occupancy (Pink bars) compared to the mock (Blue bars) at different distances downstream from the EAGs are represented in input %. Distances from EAGs on the indicated genes: Cluster H: I: +0.1–0.3 kb, II: +1.5–2 kb; Cluster PA 1–3: I: +0.5–1 kb, II: +2–3 kb, III: +4–5 kb. Error bars represent +/− standard deviations. **E**) The locations of oligonucleotides, which were used to validate Pol II pause profiles, are represented schematically.

As in our initial analysis (Figure 1A and B), both expressed and non-expressed genes were analysed to characterize Pol II pausing, we next subdivided the non-neighboring 13787 isolated genes relative to their i) 3′ Pol II pause densities and ii) expression. Pol II enrichment signals −/+4 kb around the EAGs were used for K-means clustering (Figure S1A) [45]. Average Pol II tag density −/+4 kb of EAGs was calculated (Figure S1B) and average gene density profiles, considering regions −/+4 kb upstream and downstream of the transcription unit were also generated (Figure S1C). The identified Pol II enrichment patterns in combination with gene expression data allowed us to subdivide the non-neighboring 13787 refseq genes in two clusters (Figure S1A). These data together with the analysis of the expression of the genes in the two clusters show that genes in Cluster 2 are not or very weakly expressed as compared to genes in the Cluster 1 (Figure S1D). Thus, in order to study Pol II occupancy only at transcribed genes we analysed the 3495 expressed genes (Cluster 1 in Figure S1) by re-clustering them (Figure 2). Using K-means clustering and seqMINER, a ChIP-seq data interpretation platform [45], four new clusters based upon Pol II tag densities and patterns were generated. Distinct patterns of Pol II binding profiles downstream of the EAGs can be divided as follows: narrow (Cluster H), very broad (Cluster PA1) and broad pause (Cluster PA2) (Figure 2A and B). Interestingly, gene ontology (GO) analyses of members in these categories indicated that genes in the narrow cluster are almost exclusively core histone genes, which are intronless, code for non-polyadenylated transcripts and involved in replication-dependent nucleosomal assembly (Table 1), hereafter called Cluster H. Note that the other three categories contained genes coding principally for polyadenylated (PA) transcripts, thus these clusters are called PA1-3. In the narrow peak-containing Cluster H, Pol II occupancy signals decrease very rapidly after the EAG. In contrast, in Cluster PA1 and PA2, Pol II occupancy is quite widespread and can be detected until 4–6 kb downstream from the EAGs (Figure 2B). The difference between PA1 and PA2 seems to be related to gene expression as genes belonging to Cluster PA1 are higher expressed than those belonging to Cluster PA2 (Figure 2C) which is in good agreement with their higher Pol II occupancy downstream of the EAGs (Figure 2B). Genes in the Cluster PA3, which have less (or very less) Pol II enrichment, also show detectable Pol II occupancy downstream of the EAGs (Figure 2B).

**Table 1:** Gene Ontology (GO) terms (at significant P-values), associated with the clusters represented in Figure 2.

Cluster	Number of genes	Top GO biological process	P-value
Cluster H	39	Nucleosome assembly	1.48E-012
Cluster PA1	74	Structural constituent of cytoskeleton	6.90E-003
		Translational elongation	4.68E-002
		Cell morphogenesis involved in neuron differentiation	4.68E-002
Cluster PA2	492	Translational elongation	6.15E-032
Cluster PA3	2890	Modification dependent protein catabolic process	5.10E-010
		Chromatin modification	2.01E-009

To validate the bioinformatically isolated Pol II pause patterns downstream of the EAG (shown in Figure 2A and B), we have carried out ChIP followed by quantitative PCR detection (ChIP-qPCR). To this end we have randomly chosen two genes from each cluster and designed primer pairs to detect Pol II occupancy downstream from their EAGs (Figure 2D and E). For Cluster H, which contains mainly histone genes, primers were designed about 100–300 bps and 1.5–2 kb downstream from EAGs (I and II in Figure 2D, upper most panel; Figure 2E left panel). For Clusters PA1-3 primer pairs were chosen on the selected genes to detect peaks in regions approximately 0.5–1 kb, 2–3 kb and 4–5 kb downstream of the EAGs (see I, II and III respectively in Figure 2D; Figure 2E right panel). These validation experiments confirmed the bioinformatically defined Pol II occupancy patterns in each of the four clusters. Further in good agreement with the bioinformatics analysis of ChIP-seq, on the narrow peak containing histone cluster (Cluster H), ChIP-qPCR using primer pair II (situated 1.5–2 kb from EAG) did not amplify any significant product, indicating that Pol II occupancy is rapidly dropping after the 3′end of histone genes. In contrast, on the other three clusters, ChIP-qPCR confirmed that Pol II occupancy downstream of the EAG in general could be detected on a large region, often covering 4–6 kb downstream of the EAGs. Thus, similarly to PPP (see Introduction), we interpret these relatively high Pol II binding signals downstream of genes as Pol II pausing. Moreover, these experiments suggest that Pol II pausing 3′ from genes is different on genes from which the transcribed pre-mRNA is polyadenylated (Clusters PA1 to 3), or not (Cluster H). This genome-wide observation is in good agreement with previous suggestions based on single gene analyses [40] and may suggest a possible link between Pol II pausing and the 3′ end processing of the corresponding transcripts.

### Pol II pausing downstream from the EAGs on highly expressed genes

**Figure 3:**
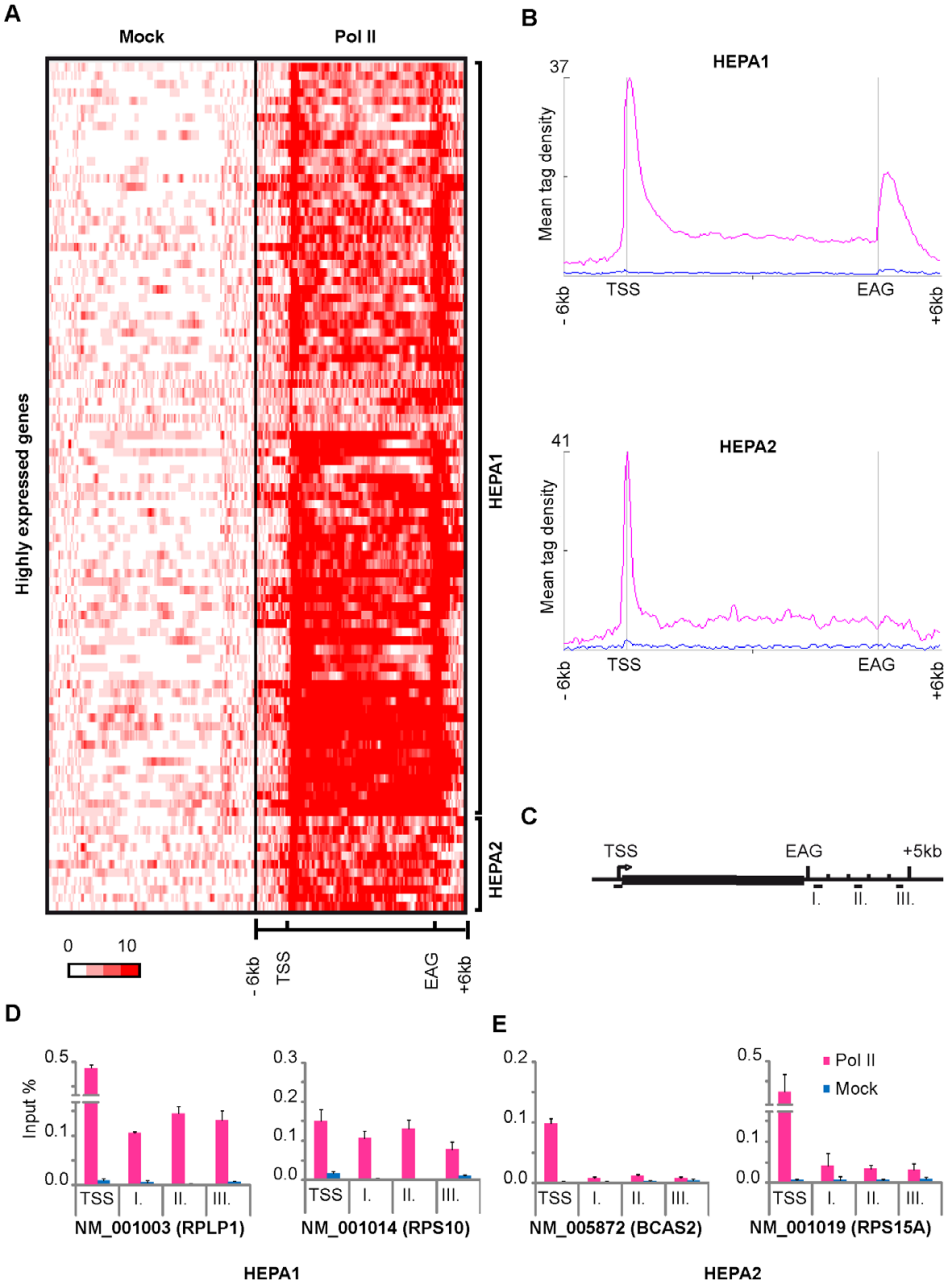
Pol II pause on highly expressed genes. K-means clustering of Mock (blue) and Pol II (pink) reads on 100 highly expressed genes from MCF7 cells (for exact gene names see Table S2) mainly generated two distinct clusters in terms of Pol II occupancy at the corresponding EAGs. **A**) Heatmap generated after the K-means clustering of Mock (Blue) and Pol II (Pink) reads in average gene body and −/+6 kb upstream and downstream of the genes. Color scale indicates the level of enrichment. **B**) Mean tag densities of Mock (Blue) and Pol II (Pink) signals on the two clusters of genes and −/+6 kb upstream and downstream of the gene body. **C**) The locations of oligonucleotides, which were designed to validate Pol II pause profile, are represented schematically. **D, E**) ChIP-qPCR validation of the ChIP-seq data for two randomly selected genes from each cluster (as indicated). Pol II occupancy (Pink bars) compared to mock (Blue bars) on the TSS and at different distances downstream from the EAGs are represented in input %. Distances from EAGs of the indicated genes: I: +0.5–1 kb, II: +2–3 kb, III: 4–5 kb. Error bars represent +/− standard deviations.

Next we analysed Pol II occupancy on 100 highly expressed poly(A)^+^ genes (HEPA), independently from the distance of the next neighboring gene(s), and found that on the majority of highly expressed genes Pol II pausing downstream of the EAG sites is similar to that described above in Cluster PA1 (see Cluster HEPA1, Figure 3A and B). However, a small subset of highly expressed genes (about 10%) have high Pol II enrichment at their TSSs, but no or very little Pol II occupancy downstream of their EAGs (Cluster HEPA2 of Figure 3A and B).

To validate Pol II occupancy on genes in Cluster HEPA1 and HEPA2 we have carried out ChIP-qPCR. To this end we have compared Pol II occupancy downstream of the EAGs on two randomly chosen highly expressed genes with high 3′ peak with those which have no or only very low 3′ peaks (Figure 3C and D). For genes belonging to each cluster, primer pairs were chosen to detect peaks both at their TSSs and in regions approximately 0.5–1 kb, 2–3 kb and 4–5 kb downstream of the EAGs (I, II and III, respectively in Figure 3E). These ChIP-qPCR experiments confirmed the absence of significant Pol II occupancy downstream of the EAGs in a small fraction of the highly expressed genes suggesting that on a smaller subset of genes Pol II pausing at the 3′ end of the genes might be differentially regulated.

### Pol II pausing downstream from the EAGs of core histone genes is different from those transcription units producing polyadenylated transcripts

**Figure 4:**
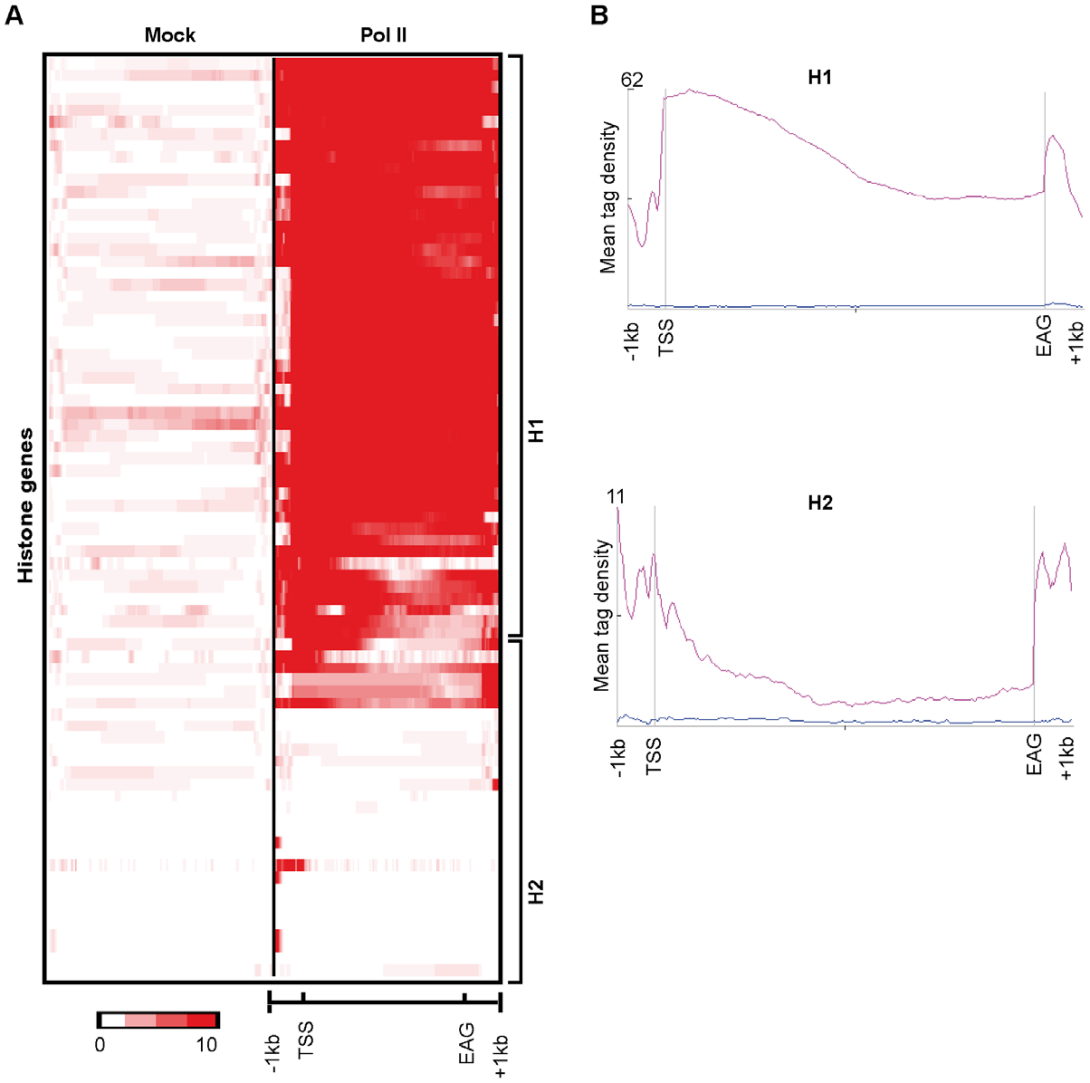
On core histone genes Pol II occupancy downstream of the EAGs is quickly dropping. Clustering of reads obtained following anti-Mock ChIP-seq and anti-Pol II ChIP-seq on all human histone genes generates two clusters. **A**) Heatmap generated after the K-means clustering of Mock and Pol II reads in average gene body and −/+1 kb upstream and downstream of the genes. Color scale indicates the level of enrichment. **B**) Mean tag densities of Mock (Blue) and Pol II (Pink) signals in two clusters of genes (H1 = core histone; H2 = variant histone) in the average gene body and −/+1 kb upstream and downstream of the genes.

Replication dependent core histone genes are intronless and coding for mRNAs that have a different 3′ processing mechanism than mRNAs transcribed from poly(A)^+^ genes. Instead of polyA tail addition, the cleavage-only mRNA 3′-end formation of core histone genes involves stem-loop formation, the U7 snRNP, hairpin-binding protein and specific components of the cleavage/poly(A) complex [46], [47], [48], [49], [50]. As described in our above analysis, looking for genes that have no neighboring genes in the 4 kb vicinity, the narrow Pol II peak-containing Cluster H contained mainly core histone genes, prompted us to map Pol II profile in and around all the known histone genes from the human genome. As core histone genes are often found in clusters we have analysed Pol II occupancy only −/+1 kb upstream and downstream of histone genes. These new K-means clustering and profiling analyses of Pol II 1 kb around all histone genes show a high Pol II enrichment throughout the gene body and a sharp drop in the Pol II occupancy 3′ of the EAGs of core histone genes (Cluster H1 of Figure 4A, B). In contrast, Pol II occupancy on genes encoding non-replication dependent histone variants (which generally have introns, are weaker expressed than core histone genes and their transcripts undergo polyadenylation) is different from the core histone genes (Cluster H2 of Figure 4A, B). On the variant histone genes we did not observe the sharp drop of Pol II occupancy downstream of the EAGs (Figure 4B) as in the core histone genes, suggesting that 3′ end processing on core histone genes involving stem-loop formation may be in favor of a rapid Pol II release. Moreover, our results further suggest that the polyadenylation of variant histone transcripts may participate in Pol II pausing on the corresponding variant histone genes before transcription termination may occur.

**Figure 5:**
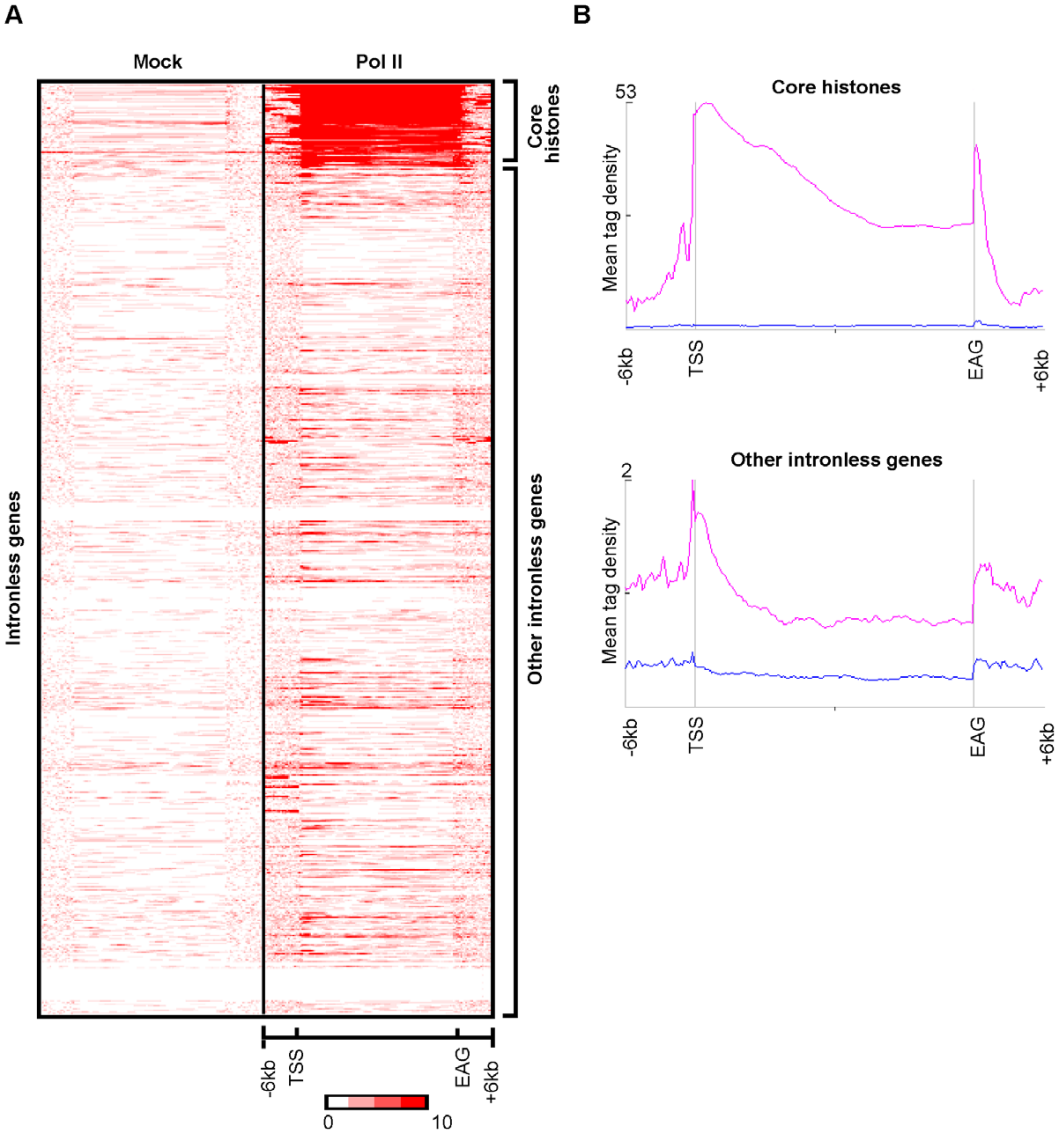
Amongst intronless genes only histone genes have narrow Pol II pause peaks downstream of the EAGs. Clustering of Mock and Pol II reads on all human intronless genes generates two clusters. **A**) Heatmap generated after the K-means clustering of Mock and Pol II reads in average gene body and −/+6 kb upstream and downstream of the genes. Color scale indicates the level of enrichment. **B**) Mean tag densities of Mock (Blue) and Pol II (Pink) in two clusters (Core histones and Other intronless genes) −/+6 kb upstream and downstream of the gene body.

As core histone genes are intronless, we have analysed Pol II occupancy on all intronless genes from the human genome to test whether the narrow Pol II pause profile downstream of the EAG was characteristic of core histone genes or, rather, a common feature of genes producing transcripts that do not undergo splicing. Interestingly, the sharp drop of Pol II occupancy occurred only downstream of EAGs of core histone genes suggesting that the narrow Pol II pausing pattern 3′ of EAGs is the characteristic of core histone genes (Figure 5). Taken together these results with the above qPCR validation results, suggest that Pol II pausing downstream from the EAGs of core histone genes is different from those transcription units producing polyadenylated transcripts.

### Differential Pol II pausing downstream from the EAGs of core histone genes and poly(A)^+^ genes is conserved between mouse and human cells and seems to be independent from the developmental stage of the cells

**Figure 6:**
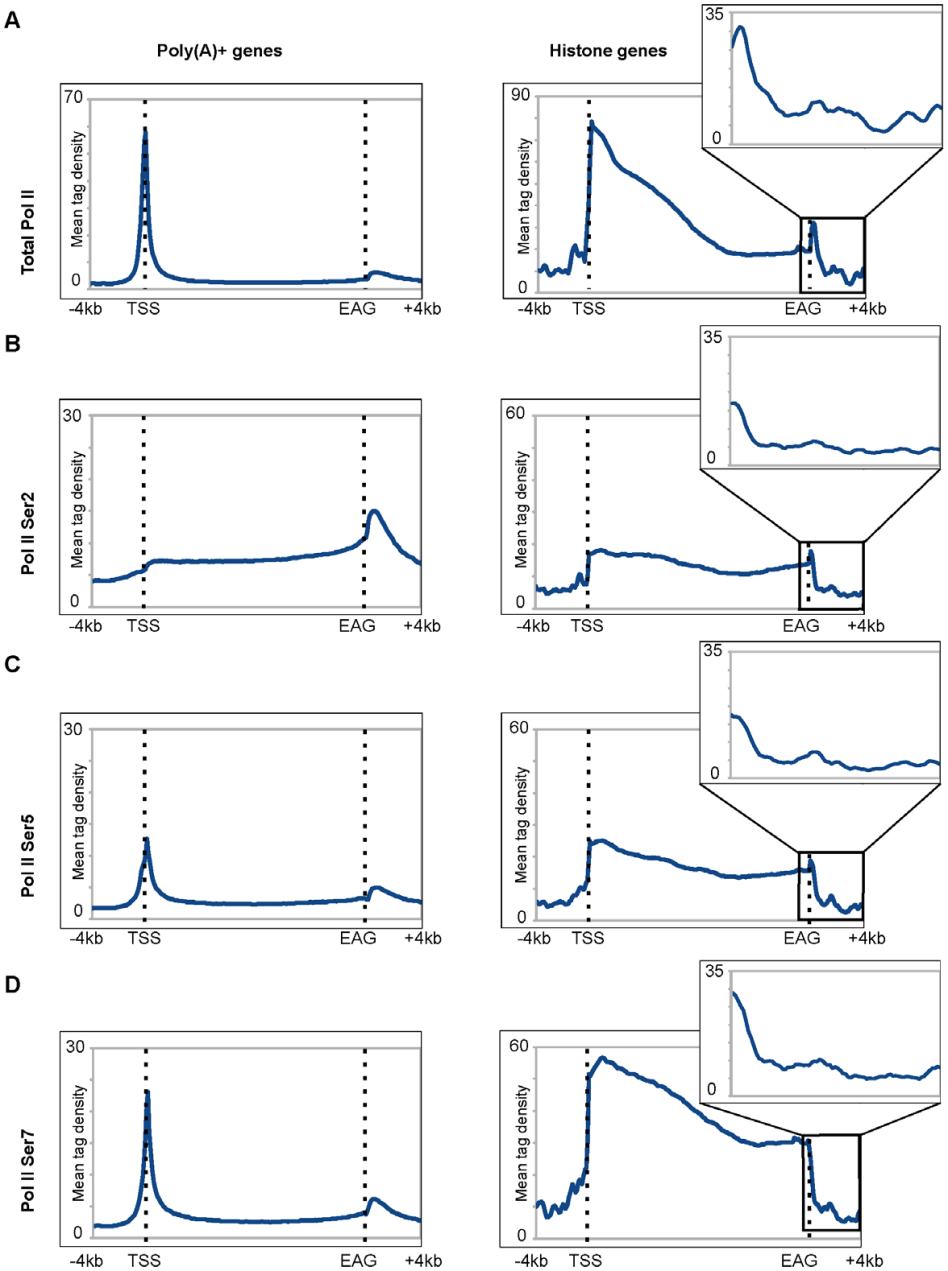
Differential Pol II pausing downstream from the EAGs is conserved between mouse and human cells and seems to be independent from the developmental stage of the cells. **A–D**) Four different published Pol II ChIP-seq data sets from mES cells [5], [43] were used to generate average gene profiles for different forms of Pol II (Total, Ser2, Ser5 and Ser7 phosphorylated form of the CTD of the largest subunit of Pol II) for Poly(A)^+^ and core Histone genes, as indicated on the top of the figure and on the left of the panels. Y-axis represents mean tag densities.

In order to further investigate the differential pattern observed for Pol II occupancy downstream of the EAGs on core histone and poly(A)^+^ genes in differentiated human cells and to analyse the state of the phosphorylation of the C-terminal domain (CTD) of the largest subunit of Pol II, we analysed four different published Pol II ChIP-seq data sets from pluripotent mouse embryonic stem cells (mESs) [5], [43]. In one of these studies, genome-wide Pol II occupancy was investigated by using the antibody, which can recognize the N-terminus of the largest subunit of Pol II (as above for human MCF7 cells), allowing monitoring Pol II independent of the phosphorylation status of its CTD (see also “Total Pol II” Figure 6A). In addition, ChIP-seq data were also available for the Ser2, Ser5 and Ser7 phosphorylated form of CTD of Pol II from mES cells [5], [43]. Using these data sets, we created two categories of genes for each form of Pol II: actively transcribed poly(A)^+^ genes and core histone genes (Figure 6). In mES cells, similarly to human cells, we observed (i) a differential Pol II pausing downstream from the EAGs (Figure 6A, “Total Pol II) and (ii) that “total” Pol II pausing at the 3′ end of histone genes is narrow as compared to the broad pause observed downstream of the expressed poly(A)^+^ genes (Figure 6A). We also investigated which form of Pol II is recruited to the 3′ end of core histone genes (Figure 6B, C and D). Our analyses show that Pol II occupancy profiles 3′ from the EAGs are very comparable between differentiated human and pluripotent mouse cells (compare Figure 1 and 2 to Figure 6A). Moreover, we find that Ser2, Ser5 and Ser7 phosphorylated forms of Pol II are present, but drop rapidly at the 3′ end of the core histone genes (Figure 6C and D). In contrast, but in good agreement with previous studies [5], [43], Ser2 phopshorylated form of Pol II is mainly present in the gene body and peaks downstream of the EAGs of poly(A)^+^ genes (Figure 6B). Taken together these results suggest that differential Pol II pausing downstream of the EAGs of either core histone or poly(A)^+^ genes is conserved in vertebrate cells and seems to be independent from the developmental stage of the cells. The observed conservation of the distinct Pol II pausing downstream from the EAGs between core histone and poly(A)^+^ genes further suggest a possible differential link between Pol II pausing and the 3′ end processing of the corresponding transcripts.

### Inhibition of polyadenylation increases Pol II occupancy downstream of the EAGs on poly(A)^+^ genes, but not on core histone genes

**Figure 7:**
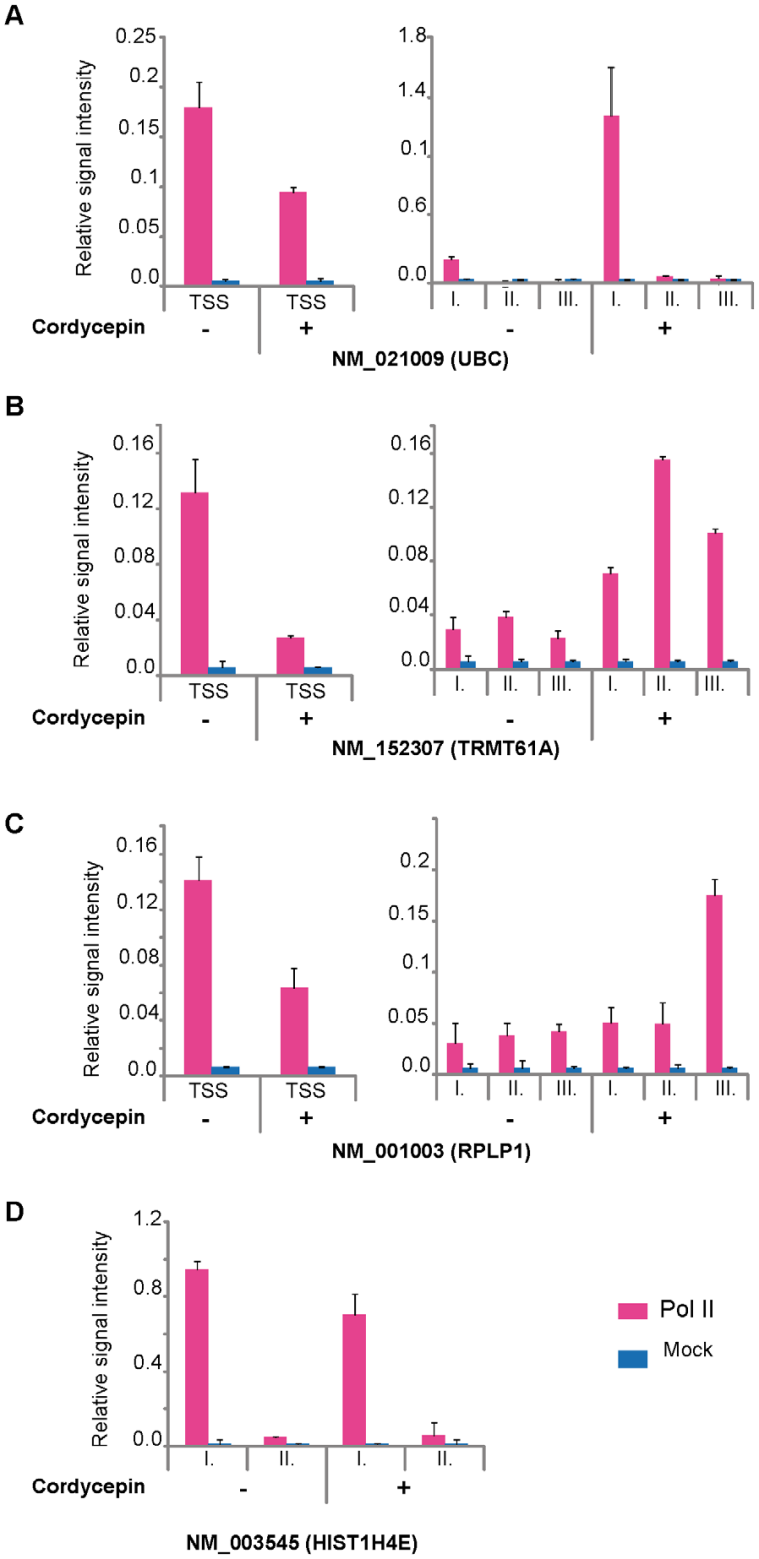
Inhibition of polyadenylation increases Pol II occupancy downstream of the EAGs on poly(A)^+^ genes, but not on core histone genes. ChIP-qPCR validation of Pol II occupancy following cordycepin treatment on poly(A)^+^ genes (**A–C**) and histone gene (**D**). Pol II occupancy (Pink bars) is compared at promoters and at different distances downstream from the EAGs before and after cordycepin treatment. Values are normalized to mock controls (Blue bars) and represented as relative signal intensity values. Distances: A, B, C: +0.5–1 kb, II: +2–3 kb, III: +4–5 kb from EAGs of the indicated poly(A)^+^ genes; D: I: +0.1–0.3 kb, II: +1.5–2 kb from EAG the indicated core histone gene. Error bars represent +/− standard deviations.

To test whether there is a functional link between Pol II occupancy downstream of the EAGs and the 3′ end processing of the corresponding transcripts we have inhibited poladenylation using cordycepin ([51] and refs therein), and tested by ChIP-qPCR whether we can observe a change in Pol II occupancy downstream of the EAGs on poly(A)^+^ genes when compared to core histone genes. To test the effect of polyadenylation inhibition, MCF7 cells were either not treated, or treated for 3 hours with cordycepin, as described earlier [51]. Following the treatment, cells were subjected to ChIP-qPCR analysis as described above. We observed that on the tested poly(A)^+^ genes inhibition of polyadenylation increased Pol II occupancy downstream of the EAGs (Figure 7 A, B and C; right panels). In contrast, on the tested core histone gene the cordycepin treatment had no significant effect on the Pol II signal at the 3′end of the EAG (Figure 7 D). These observations show that on poly(A)^+^ genes there is a link between polyadenylation and the broad Pol II pausing downstream of the EAGs and further suggest that Pol II pausing 3′ of the EAGs, and consequent termination, may undergo different types of regulation depending on the 3′ end processing mechanisms of the transcripts.

Interestingly, the inhibition of polyadenylation by cordycepin not only increased Pol II occupancy downstream of the EAGs of poly(A)^+^ genes, but reduced Pol II occupancy at the TSSs of the genes tested (Figure 7A, B and C; left panels). This observation is in good agreement with previous findings suggesting a functional link between transcription initiation and termination by Pol II [52]. Note that at the TSSs of core histone genes the q-PCR reactions could not be carried out because the sonicated and ChIP-ed DNA fragments are longer (500–1500 bps) than the distance would be between the TSS of core histone genes and their EAGs (300–500 bp), where the first q-PCR primer pair has been designed (see Figure 7D).

## Discussion

### Pol II pausing on long regions downstream of the EAGs is a common feature of genes producing polyadenylated transcripts

Various RNA-processing events have been shown to occur cotranscriptionally (reviewed in [14], [24], [28], [53], [54]). Despite its apparent simplicity, the mechanism of Pol II termination is not yet well understood. Moreover, different terminator sequences in the downstream regions of EAGs and different transcription termination mechanisms coupling 3′ end processing and termination exists (see Introduction). However, in the case of core histone genes the link between the lack of Pol II pausing and pre-mRNA 3′ end processing is less well characterized.

By analyzing genome-wide Pol II occupancy together with Gro-seq data [42] in human MCF7 cells and Pol II occupancy in mouse ES cells we report that on most of the expressed human and mouse poly(A)^+^ genes transcriptionally active Pol II pausing downstream of EAGs is a common event. On these genes, Pol II occupancy often covers a very long region, as ChIP signals can be mapped up to 4–6 kb downstream of the EAGs. The fact that majority of the expressed poly(A)^+^ genes have significant Pol II occupancy throughout a long region after the 3′ of the genes and that core histone genes have a very sharp drop of Pol II occupancy at 3′ of their EAGs, suggests that the different 3′ processing regulatory mechanisms influence the residency time of Pol II downstream of the EAGs. These differential Pol II occupancy mechanisms 3′ of the distinct gene categories seem to be conserved in vertebrates and not influenced by the differentiation state of the cells.

According to a recent report, expressed genes are linked to different specialized transcriptional factories and the size of the “factory” depend on the strength of the expression of the genes connected to these sites of transcription [55]. Thus, it is conceivable that different transcription factories might have a link with the different pause patterns observed in this analysis. In agreement, poly (A)^+^ genes belonging to the broad category are higher expressed than those belonging to the very broad category (Figure 2). Note, however, that in terms of DNA sequence, amongst the downstream regions of poly(A)^+^ genes belonging to the very broad and broad categories we could not identify any significant differences and we do not know whether “allosteric-/anti-terminator” or “torpedo” model type termination mechanisms, or a hybrid of the two mechanisms, would play a role.

Our genome-wide results also suggest that the pausing step downstream of the EAGs reflects a slowing down of the Pol II elongation complex. It seems that on genes that produce polyadenylated transcripts Pol II binding downstream of EAGs is in general different than during transcription in the gene body in terms of detectable Pol II occupancy. Moreover, in most of the cases after the cleavage event at the polyadenylation site, transcriptionally active Pol II can be detected for a long time and distance on the DNA template. In good agreement, all the transcripts identified by Gro-seq are mapping in the sense orientation 3′ from the EAGs, suggesting that they are produced by Pol II molecules that continue to transcribe after having finished the pre-mRNAs. In this respect it is interesting that inhibition of polyadenylation increased Pol II occupancy downstream of EAGs on poly(A)^+^ genes suggesting that defective polyadenylation can signal to the terminating Pol II, to slow down. Such a signaling may be necessary to reduce transcription on poly(A)^+^ genes, where polyadenylation would be defective. The increase of Pol II residency time 3′ of poly(A)^+^ genes in turn may reduce the amount of Pol II that can be released from each gene to enter in a new reinitiation cycle. This hypothesis is in good agreement with our findings showing that inhibition of polyadenylation reduces Pol II occupancy at the TSSs of the tested genes (Figure 7). Thus, Pol II pausing downstream of the EAGs may be implicated in a feedback regulation of 3′ processing of the nascent transcripts, where Pol II 3′ pausing would be controlled by the completeness of the polyadenylation of the pre-mRNA and thus, may prevent immediate Pol II release from the gene [16], [25], [56]. Consequently, longer Pol II pausing in regions downstream of the EAGs may play a role in the establishment of nuclear pools of Pol II that can be engaged in new rounds of transcription. These observations further point towards a link between transcription initiation and termination as previously suggested [57], [58]. In agreement with this hypothesis, several general transcription factors, such as TFIIB, TFIID, TFIIH and the Mediator complex, have been described in linking transcription initiation and Pol II termination [59], [60], [61], [62], [63]. Moreover, these studies also hypothesized that on actively transcribed genes looping may occur [57], [58], [64]. The finding that a small subset of highly expressed poly(A)^+^ genes exist with no, or weak, Pol II pause downstream of the EAGs may suggest that on these genes transcription initiation and termination are not linked or that on these subset of highly expressed (often ribosomal protein coding) genes a very efficient Pol II recycling is required.

### On core histone genes Pol II occupancy is dropping sharply downstream of the EAGs

Core histone genes are generally small in size, intronless and encoding for transcripts that do not undergo polyadenylation. The 3′ end of core histone mRNAs is formed by a cleavage reaction between the stem–loop and the purine-rich sequence [65], with transcription continuing for at least a few hundred nucleotide past the 3′ end of the mRNA [66]. In contrast to poly(A)^+^ genes, at core histone genes Pol II occupancy downstream of the EAGs quickly declines (Figure 2 and 4). Such short Pol II pause may be of high importance since often core histone genes can be found in clusters, separated by only short distances (i.e. 0.8–1 kb) and thus, Pol II occupancy has to decline rapidly to prevent Pol II from running into the neighboring gene in the cluster. Our observations suggest that in spite the fact that also common core cleavage factors are required for processing of core histone and polyadenylated pre-mRNAs [67], there are important differences remaining in the way how Pol II is released from the different templates coding for the two types of transcripts during transcription termination. Thus, as the 3′ processing of core histone transcripts are carried out by a distinct machinery than those transcripts generated from poly(A)^+^ genes, it is conceivable that the differential Pol II pause profiles observed on core histone genes versus poly(A)^+^ genes represents a differential interaction between Pol II and the two different 3′ processing machineries. This idea is also corroborated by the functional studies, which showed that inhibition of polyadenylation increased Pol II occupancy 3′ of the EAGs of poly(A)^+^ genes, while it had no significant effect on Pol II drop at the 3′ end of the core histone genes.

Our findings are in good agreement with previous reports describing a connection between polyadenylation and Pol II pause followed by transcription termination (reviewed in [24], [39]) and in addition suggests that this connection is differently regulated on core histone genes, where 3′ processing of the transcripts is distinct from other protein coding genes. It seems that Pol II release on histone genes is an actively regulated and quick event. It may be in relation with the high transcription rate and the possible recycling of Poll II on these genes for quick transcription re-initiation. Interestingly, histone variant genes which are different from core histone genes, because they contain introns and their encoded transcripts are polyadenylated, show extended Pol II pause downstream of their EAGs in contrast to core histone genes, further suggesting that polyadenylation might have a role in the Pol II slow down and hence a longer pause.

Taken together, our study suggests that in mammalian cells Pol II pausing downstream of the EAGs and mRNA 3′ processing are not independent, but linked. Thus, it seems that pausing of Pol II 3′ from the EAG is part of transcription regulatory mechanisms at different type of genes.

## Materials and Methods

### Chromatin immunoprecipitation

Human MCF7 cells (obtained from American Type Culture Collection; ATCC; reference number HTB-22) were grown up to 85–90% confluence, washed with PBS, and cross-linked with 1% formaldehyde for 20 min at room temperature. The reaction was stopped with 0.5 M glycine, and cells were washed three times with ice-cold PBS supplemented with 0.5 mM phenylmethylsulfonyl fluoride (PMSF), scraped, and resuspended in swelling buffer (25 mM HEPES, pH 7.8, 1.5 mM MgCl_2_, 10 mM KCl, 0.1% NP-40, 1 mM dithiothreitol [DTT], 0.5 mM PMSF, protease inhibitor cocktail [PIC], Amersham). Cells were broken with a Dounce homogenizer, and the nuclear fraction was resuspended in sonication buffer (50 mM HEPES, pH 7.8, 140 mM NaCl, 1 mM EDTA, 1% Triton X-100, 0.1% Na-deoxycholate, 1% sodium dodecyl sulfate [SDS], 0.5 mM PMSF, PIC). The chromatin was sonicated with a Bioruptor (Diagenode) sonicator into 100–500-bp fragments and centrifuged to avoid any remaining cell debris.

From the supernatant, 30 µg chromatin diluted with sonication buffer (without SDS) up to 1 ml (0.05% SDS concentration) was used for one IP. For ChIP-seq 5 samples were added together. Protein G Sepharose beads were washed and blocked with sonication buffer containing cold-water fish skin gelatin (SIGMA) and yeast tRNA. Chromatin samples were pre-cleared with unblocked beads at 4°C, for 2 hours. The precleared chromatin samples were rotated overnight at 4°C with the Pol II antibody (Santa Cruz, H-224X), and then blocked beads were added for 2 hours to the samples to pull down specific protein-DNA complexes. After immunoprecipitation, samples were washed two times at 4°C with the following buffers: twice with Sonication buffer (without SDS), twice with buffer A (50 mM HEPES, pH 7.8, 500 mM NaCl, 1 mM EDTA, 1% Triton X-100, 0.1% Na-deoxycholate, 0.1% SDS, 0.5 mM PMSF, PIC), twice with buffer B (20 mM Tris, pH 8, 1 mM EDTA, 250 mM LiCl, 0.5% NP-40, 0.5% Na-deoxycholate, 0.5 mM PMSF, PIC), and finally twice with Tris-EDTA buffer (10 mM Tris, pH 8, 1 mM EDTA). Bound fraction of the chromatin was eluted with 2×100 µl of elution buffer (50 mM Tris, pH 8, 1 mM EDTA, 1% SDS) at 65°C for 2×10 min and elutions were pooled. RNase A treatment (5 µg/ml), and reverse cross-linking (125 mM NaCl) was carried out at 65°C overnight. Elutions were finally incubated with proteinase K. DNA was phenol-chloroform extracted and precipitated by ethanol. Validation of the ChIP was performed by quantitative PCR (qPCR) analysis using a Roche LightCycler 480 with Sybr green (Roche) master mix.

As a negative control, we immunoprecipitated the cross-linked material with a yeast antibody. The ChIP experiments were repeated at least twice, and all the qPCR reactions were done in triplicates.

To inhibit polyadenylation human MCF7 cells were grown up to 85–90% confluence and the medium was changed with fresh one containing cordycepin (Sigma, 50 µg/ml final concentration) [51]. After three hours of treatment, ChIP was carried out as described above.

### Solexa high throughput sequencing

Sample preparation was performed as described by the manufacturer. The 32 base pair tags generated from Illumina/Solexa were mapped to the human genome Build 36.1 (UCSC hg18) using the eland program allowing two mismatches. Only sequences that mapped uniquely to the genome with maximum of two mismatches were used for further analysis. We obtained 10.9 and 4.5 millions uniquely mapped reads for Pol II and Mock, respectively. Using the liftover tool from UCSC (www.ucsc.org), tags were mapped onto the human genome hg19. The obtained Pol II ChIP-seq data from human MCF7 cells was deposited in GEO under GSE34001number.

### Validation of ChIP-Seq by comparison to real-time PCR

qPCR was performed by Roche LightCycler 480 with Sybr green (Roche) master mix. The sequences of the oligonucleotides are available upon request.

### Genome Annotations

Genome annotations were downloaded from the UCSC Genome Browser (www.ucsc.org), human genome Build 37 (hg19 assembly). Gene definitions were given by the refseq genes [68] track. For the analysis mentioned in the paper, we have considered only those refseq genes which are reviewed and validated.

### Heatmap and Clustering of Pol II patterns

For all the refseq genes, which were analysed, we extracted the tag density in a 4 kb windows surrounding the EAG and surrounding the gene body using the program seqMINER which generates heatmap as well as the profiles [45]. The sequenced ChIP-seq reads represent only the end of each immunoprecipitated fragments instead of the precise protein-DNA binding sites. To illustrate the entire DNA fragment, basically before analysis, 3′ end of each ChIP-seq read was extended to 200 bp in the direction of the reads. In order to get an average gene profile of ChIP-seq tags, genes from its start (5′ end) to end (3′ end) [according to refseq annotation] were averaged in a 50 bp window. While doing this analysis, the strand orientation is taken in account in order to orientate all analysed features in the same direction. Pol II tag densities were subjected to K-means clustering in order to organize or cluster genes in a same group based upon similar tag enrichment within a defined region. In K-means clustering, number of clusters is fixed a priori and hence we define the number of clusters based upon the tag enrichment and patterns of Pol II.

### Microarray Expression Data

Cel files of MCF7 cells were downloaded from Gene Expression Omnibus (GSE18912, http://www.ncbi.nlm.nih.gov/geo/). In this experiment, MCF7 cells were tested as five replicates using Affymetrix U133 Plus 2.0 arrays. The cel files were normalized by gcrma method [69], and calculations were performed using R (http://www.r-project.org/). The expression level for each probeset was calculated as the mean of the five replicates. While processing the data, we applied the following filters. Firstly, we excluded probesets assigned to several genes localized on different places on the genome to avoid annotation artifacts. Secondly, we eliminated all low intensity probesets where signal cannot be distinguished from noise. These filters allowed us to obtain curated list of expressed probesets. A single relative expression value was computed for each gene and based upon relative expression values we selected lists of highly expressed genes.

### Gene Ontology

Enrichment of GO categories was determined using WebGestalt software [70] and functional categories with stringent p-value (p-value<0.01) have been considered for the analysis.

## Supporting Information

Figure S1 Showing genome-wide Pol II occupancy on “non-neighboring” human genes.
(DOC)

Table S1 List containing 500 highly expressed genes from MCF7 cells used in the analysis in **Figure 1C**.
(DOC)

Table S2 List containing 100 highly expressed genes from MCF7 cells used in **Figure 3**.
(DOC)
