# Supplementary material for: RNA Polymerase II Pausing Downstream of Core Histone Genes Is Different from Genes Producing Polyadenylated Transcripts
Source: PLoS One. 2012 Jun 11;7(6):e38769. doi: 10.1371/journal.pone.0038769 (PMC3372504; doi:10.1371/journal.pone.0038769)
Supplement: Figure S1 — Showing genome-wide Pol II occupancy on “non-neighboring” human genes. (DOC) [file pone.0038769.s001.doc]

**Supporting Information to Figure S1:**

**RNA polymerase II pausing downstream of core histone genes is different from genes producing polyadenylated transcripts**

**Krishanpal Anamika^1,2,3^, Akos Gyenis^1,3^, Laetitia Poidevin^2^, Olivier Poch^2^,**

**and Làszlò Tora^1,4^**

^1^Department of Functional Genomics and Cancer, ^2^Department of Structural Biology and Genomics, Institut de Génétique et de Biologie Moléculaire et Cellulaire (IGBMC), CNRS UMR 7104, INSERM U 964, Université de Strasbourg, 1 Rue Laurent Fries, 67404 Illkirch Cedex, France

^3^Equal first authors

^4^Corresponding author. Tel: +33 388653444, Fax: +33 388653201, Email: [laszlo@igbmc.fr](mailto:laszlo@igbmc.fr)

Running title: Differential 3’ Pol II pausing

**Key words:** ChIP-sequencing, genome-wide mapping, global run-on and sequencing (Gro-seq), 3’ end of gene, Pol II pause, core histone genes, transcription termination, MCF7 cells, transcription start sites, polyadenylation.


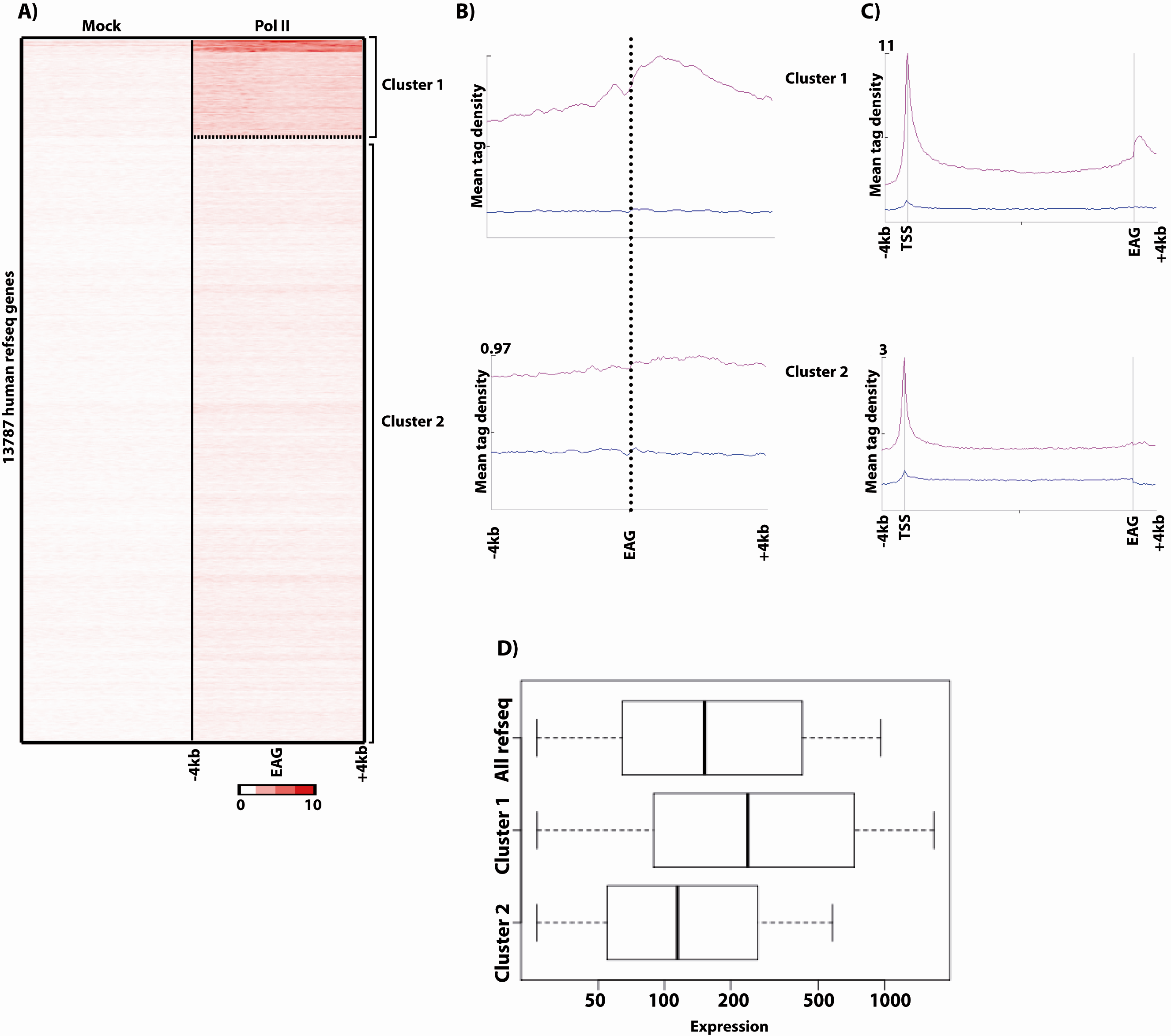


**Figure S1: Genome-wide Pol II occupancy on “non-neighboring” human genes**

Clustering of Mock and Pol II reads on 13787 non-redundant human refseq genes, which do not have genes in the -/+4 kb neighborhood, generates two clusters based upon Pol II enrichment. Number of genes (or n) in each cluster is: Cluster 1, n=3495; Cluster 2, n=10292. **A**) Heatmap generated after K-means clustering of Mock and Pol II reads in the region -/+4 kb upstream and downstream of the EAG. Color scale indicates the level of enrichment. **B**) Mean tag densities of Mock (Blue) and Pol II (Pink) reads on genes from each of the two clusters -/+ 4 kb upstream and downstream of the EAG. **C**) Mean tag densities of Mock (Blue) and Pol II (Pink) reads on genes from each of the two clusters -/+4kb upstream and downstream of the gene. D) Box plot showing microarray expression range of all the refseq genes from MCF7 cells and of all the genes in each of the two clusters.
