# Supplementary material for: RNA Polymerase II Pausing Downstream of Core Histone Genes Is Different from Genes Producing Polyadenylated Transcripts
Source: PLoS One. 2012 Jun 11;7(6):e38769. doi: 10.1371/journal.pone.0038769 (PMC3372504; doi:10.1371/journal.pone.0038769)
Supplement: Table S1 — List containing 500 highly expressed genes from MCF7 cells used in the analysis in Figure 1C . (DOC) [file pone.0038769.s002.doc]

**Supporting Information to Table S1:**

**RNA polymerase II pausing downstream of core histone genes is different from genes producing polyadenylated transcripts**

**Krishanpal Anamika^1,2,3^, Akos Gyenis^1,3^, Laetitia Poidevin^2^, Olivier Poch^2^,**

**and Làszlò Tora^1,4^**

^1^Department of Functional Genomics and Cancer, ^2^Department of Structural Biology and Genomics, Institut de Génétique et de Biologie Moléculaire et Cellulaire (IGBMC), CNRS UMR 7104, INSERM U 964, Université de Strasbourg, 1 Rue Laurent Fries, 67404 Illkirch Cedex, France

^3^Equal first authors

^4^Corresponding author. Tel: +33 388653444, Fax: +33 388653201, Email: [laszlo@igbmc.fr](mailto:laszlo@igbmc.fr)

Running title: Differential 3’ Pol II pausing

**Key words:** ChIP-sequencing, genome-wide mapping, global run-on and sequencing (Gro-seq), 3’ end of gene, Pol II pause, core histone genes, transcription termination, MCF7 cells, transcription start sites, polyadenylation.

**Table S1: List containing 500 highly expressed genes from MCF7 cells used in Figure 1C.**

NM_001035267

NM_021109

NM_001101

NM_000224

NM_022551

NM_001021

NM_002046

NM_000973

NM_000989

NM_003225

NM_001014

NM_000976

NM_001006

NM_000994

NM_000972

NM_000984

NM_001030

NM_001402

NM_001402

NM_001540

NM_001002

NM_001030001

NM_001005

NM_001000

NM_001017977

NM_000661

NM_001011

NM_012423

NM_006597

NM_001032393

NM_000980

NM_000968

NM_002952

NM_001017977

NM_021009

NM_007209

NM_001020

NM_018955

NM_032704

NM_003295

NM_001007

NM_001017963

NM_000970

NM_001016

NM_000982

NM_000972

NM_021130

NM_001017

NM_001614

NM_001005472

NM_006082

NM_000971

NM_001028

NM_001113201

NM_001024

NM_000985

NM_000986

NM_001023

NM_001011

NM_004374

NM_021019

NM_001002

NM_001099285

NM_001022

NM_001003

XM_001717628

NM_002106

NM_001152

NM_001009

NM_007104

NM_001404

NM_001025

NM_000998

NM_001026

NM_005324

NM_000995

NM_006088

NM_000967

NM_001037738

NM_000988

NM_033296

NM_001015

NM_001018

NM_002273

NM_003096

NM_005517

NM_006098

NM_000981

NM_000977

NM_005507

NM_006401

NM_004597

NM_030979

NM_000979

NM_004152

NM_001010

NM_001029

NM_007104

NM_178014

NM_001033930

NM_001135239

NM_000034

NM_006325

NM_000269

XR_016455

NM_002107

NM_000991

NM_022340

NM_005872

NM_004965

NM_021103

NM_001011724

NM_001019

NM_001997

NM_002266

NM_001688

NM_001144944

NM_001012

NM_001001977

NM_001416

NM_007355

NM_000990

NM_006601

NM_002032

NM_000975

NM_002128

NM_001144944

NM_001743

NM_005003

NM_000993

NM_012423

NM_006408

NM_001001

NM_001037663

XR_015348

NM_014814

NM_001568

NM_001025070

NM_004547

NM_001079539

NM_014380

NM_016139

NM_000992

NM_001002295

NM_001040034

NM_032747

NM_001288

NM_002948

NM_001037808

NM_000969

NM_001001973

NM_002415

NM_006086

NM_001961

NM_002574

NM_001034996

NM_002306

NM_001127393

NM_001636

NM_014670

NM_001008393

NM_004074

NM_002635

NM_001025070

NM_001099285

NM_001130089

NM_002791

NM_015161

NM_000997

NM_001034996

NM_005620

NM_001024666

NM_001004

NM_001079862

NM_016404

NM_020685

NM_001005849

NM_014399

NM_006826

NM_002156

NM_006431

NM_001007553

NM_002568

NM_025075

NM_006476

NM_001861

NM_001135592

NM_006013

NM_021129

NM_000516

XR_016751

NM_002802

NM_002568

NM_005005

NM_002489

NM_002157

NM_001003395

NM_007208

NM_015933

NM_001863

NM_012073

NM_001878

NM_002136

NM_002966

NM_000999

NM_018997

NM_022731

NM_005213

NM_001031

NM_001031827

NM_003792

NM_001135699

NM_001127711

NM_001099406

NM_006755

NM_002796

NM_005022

NM_001144831

NM_015938

NM_003756

NM_002629

NM_013230

NM_024051

NM_001115156

NM_001102667

NM_001098576

NM_001008709

NM_014300

NM_007100

NM_020169

NM_003981

NM_001122821

NM_006234

NM_000903

NM_000983

NM_006708

NM_001013

NM_032477

NM_014176

NM_000454

NM_002654

NM_001145355

NM_004859

NM_031407

NM_006280

NM_001008800

NM_001040874

NM_000126

NM_002893

NM_003094

NM_001005207

NM_001123377

NM_001099432

NR_003038

NM_021029

NM_002805

NM_004252

NM_001130053

NM_001034

NM_001143985

NM_000269

NM_001037637

NM_000100

NR_002599

NM_006004

NM_003134

NM_001135771

NM_000996

NM_001143937

NM_014463

NM_002354

NM_001011546

NM_002792

NM_001025205

NM_006791

NM_005719

NM_005345

NM_006670

NM_001134493

NM_005731

NM_001037494

NM_002276

NM_002793

NM_003091

NM_001867

NM_007019

NM_004642

NM_015622

NM_014402

NM_016091

NM_001009570

NM_001035247

NM_002140

NM_052886

NM_014501

NM_005804

NM_005340

NM_002790

NM_005749

NM_000978

NM_002161

NM_004373

NM_014670

NM_031210

NM_001136232

NM_014713

NM_004450

NM_001130440

NM_001866

NM_020188

NM_004231

NM_001428

NM_006156

NM_002624

NM_003016

NM_003329

NM_006304

NM_005801

XM_001723401

NM_003542

NM_001130725

NM_001537

NM_001034833

NM_001007

NM_006294

NM_152573

NM_005720

NM_006694

NM_022075

NM_016039

NM_001033085

NM_003161

NM_005870

NM_004044

NM_001142418

NM_006003

NM_001958

NM_015702

NM_004541

NM_006930

NM_004499

NM_001916

NM_006430

NM_004559

NM_004396

NM_001826

NM_001002857

NM_018838

NM_005347

NM_001641

NM_016417

NR_003604

NM_004607

NM_004360

NM_001827

NM_007173

NM_018648

NM_001320

NM_015965

NM_015414

NM_006319

NM_004515

NM_002137

NM_002165

NM_001039847

NM_004092

NM_001001790

NM_031287

NM_032014

NM_005805

NM_003761

NM_003095

NM_018840

NM_001127192

NM_020151

NM_001134693

NM_002710

NM_001145408

NM_004552

NM_001130688

NM_001042467

NM_004069

NM_004462

NM_024571

NM_001037808

NM_001358

NM_001002258

NM_002947

NM_001001937

NM_001536

NM_004339

NM_030938

NM_016395

NM_012112

NM_001002244

NM_001098616

NM_013234

NM_000402

NM_177925

NM_004766

NM_001126103

NM_004175

NM_000971

NM_144998

NM_024096

NM_001539

NM_006819

NM_020300

NM_004905

NM_001018108

NM_001033088

NM_018976

NM_003404

NM_020810

NM_006908

NM_002623

NM_001012456

NM_014740

NM_006888

NM_001686

NM_000075

NM_007231

NM_001969

NM_015462

NM_001008495

NM_001436

NM_004219

NM_001006935

NM_001003696

NM_006815

NM_002812

NM_001518

NM_001003785

NM_001029989

NM_020232

NM_003620

NM_001099645

NM_003769

NM_002810

NM_014341

NM_001865

NG_008063

NM_006936

NM_002097

NM_002874

NM_016070

NM_003375

NM_001145549

NM_014886

NM_002628

NM_000146

NM_007108

NM_004545

NM_002799

NM_005916

NM_000942

NM_002818

NM_018407

NM_002876

NM_001017978

NM_006519

NM_007008

NM_001127228

NM_001130851

NM_005381

NM_024573

NM_001077442

NM_002804

NM_000169

NM_007285

NM_014623

NM_016497

NM_001007793

NM_006409

NM_198976

NM_000992

NM_012479

NM_001664

NM_018837

NM_001139441

NM_003859

NM_002720

NM_016068

NM_003017

NM_032574

NM_080593

NM_006142

NM_004553

NM_021102

NM_000175

NM_005345

NM_032111

NM_003372

NM_015932

NM_018269

NM_001025242

NM_002524

NM_001040437

NM_002787

NM_001005413
