# Supplementary material for: RNA Polymerase II Pausing Downstream of Core Histone Genes Is Different from Genes Producing Polyadenylated Transcripts
Source: PLoS One. 2012 Jun 11;7(6):e38769. doi: 10.1371/journal.pone.0038769 (PMC3372504; doi:10.1371/journal.pone.0038769)
Supplement: Table S2 — List containing 100 highly expressed genes from MCF7 cells used in Figure 3 . (DOC) [file pone.0038769.s003.doc]

**Supporting Information to Table S2:**

**RNA polymerase II pausing downstream of core histone genes is different from genes producing polyadenylated transcripts**

**Krishanpal Anamika^1,2,3^, Akos Gyenis^1,3^, Laetitia Poidevin^2^, Olivier Poch^2^,**

**and Làszlò Tora^1,4^**

^1^Department of Functional Genomics and Cancer, ^2^Department of Structural Biology and Genomics, Institut de Génétique et de Biologie Moléculaire et Cellulaire (IGBMC), CNRS UMR 7104, INSERM U 964, Université de Strasbourg, 1 Rue Laurent Fries, 67404 Illkirch Cedex, France

^3^Equal first authors

^4^Corresponding author. Tel: +33 388653444, Fax: +33 388653201, Email: [laszlo@igbmc.fr](mailto:laszlo@igbmc.fr)

Running title: Differential 3’ Pol II pausing

**Key words:** ChIP-sequencing, genome-wide mapping, global run-on and sequencing (Gro-seq), 3’ end of gene, Pol II pause, core histone genes, transcription termination, MCF7 cells, transcription start sites, polyadenylation.

**Table S2: List containing 100 highly expressed genes from MCF7 cells used In Figure 3**.

| NM_001035267 |
| --- |
| NM_021109 |
| NM_001101 |
| NM_000224 |
| NM_022551 |
| NM_002046 |
| NM_000973 |
| NM_000989 |
| NM_003225 |
| NM_001014 |
| NM_000976 |
| NM_001006 |
| NM_000994 |
| NM_000972 |
| NM_000984 |
| NM_001030 |
| NM_001402 |
| NM_001540 |
| NM_001002 |
| NM_001030001 |
| NM_001005 |
| NM_001000 |
| NM_001017977 |
| NM_000661 |
| NM_001011 |
| NM_012423 |
| NM_006597 |
| NM_000980 |
| NM_000968 |
| NM_002952 |
| NM_021009 |
| NM_007209 |
| NM_001020 |
| NM_018955 |
| NM_032704 |
| NM_003295 |
| NM_001007 |
| NM_001017963 |
| NM_000970 |
| NM_001016 |
| NM_000982 |
| NM_021130 |
| NM_001017 |
| NM_001614 |
| NM_001012321 |
| NM_006082 |
| NM_000971 |
| NM_001028 |
| NM_001113201 |
| NM_001024 |
| NM_000985 |
| NM_000986 |
| NM_001023 |
| NM_004374 |
| NM_021019 |
| NM_001099285 |
| NM_001022 |
| NM_001003 |
| NM_001416 |
| NM_001152 |
| NM_001009 |
| NM_007104 |
| NM_001404 |
| NM_001025 |
| NM_000998 |
| NM_001026 |
| NM_007355 |
| NM_000995 |
| NM_006088 |
| NM_000967 |
| NM_001037738 |
| NM_000988 |
| NM_033296 |
| NM_001015 |
| NM_001018 |
| NM_002273 |
| NM_005517 |
| NM_006098 |
| NM_000981 |
| NM_000977 |
| NM_005507 |
| NM_006401 |
| NM_000979 |
| NM_001010 |
| NM_001029 |
| NM_178014 |
| NM_001033930 |
| NM_001135239 |
| NM_000034 |
| NM_006325 |
| NM_000991 |
| NM_005872 |
| NM_004965 |
| NM_021103 |
| NM_001019 |
| NM_001997 |
| NM_002266 |
| NM_001688 |
| NM_001144944 |
| NM_001012 |
